# Chymase Inhibitor as a Novel Therapeutic Agent for Non-alcoholic Steatohepatitis

**DOI:** 10.3389/fphar.2018.00144

**Published:** 2018-02-21

**Authors:** Shinji Takai, Denan Jin

**Affiliations:** Department of Innovative Medicine, Graduate School of Medicine, Osaka Medical College, Takatsuki, Japan

**Keywords:** angiotensin II, chymase, fibrosis, inflammation, inhibitor, matrix metalloproteinase-9, non-alcoholic steatohepatitis, transforming growth factor-β

## Abstract

Non-alcoholic steatohepatitis (NASH) is characterized by inflammation and fibrosis, in addition to steatosis, of the liver, but no therapeutic agents have yet been established. The mast cell protease chymase can generate angiotensin II, matrix metalloproteinase-9 and transforming growth factor-β, all of which are associated with liver inflammation or fibrosis. In animal models of NASH, augmented chymase has been observed in the liver. In histological analysis, chymase inhibitor prevented hepatic steatosis, inflammation, and fibrosis. Chymase inhibitor also attenuated the augmentation of angiotensin II, matrix metalloproteinase-9, and transforming growth factor-β observed in the liver of NASH. Oxidative stress, inflammatory markers, and collagen were attenuated by chymase inhibition. Moreover, chymase inhibitor showed a mitigating effect on established NASH, and survival rates were significantly increased by treatment with chymase inhibitor. In this review, we propose that chymase inhibitor has potential as a novel therapy for NASH.

## Introduction

Non-alcoholic fatty liver disease (NAFLD) has been recognized as the most common form of liver disease (Angulo, 2002; Clark et al., 2002). Non-alcoholic steatohepatitis (NASH) mimics alcoholic hepatitis despite the absence of a history of drinking (Ludwig et al., 1980). NAFLD and NASH are associated with metabolic syndrome resulting from obesity, insulin resistance, hyperlipidemia, and hypertension. NAFLD is considered to be the most common liver disease and typically presents as simple hepatic steatosis (Tiniakos et al., 2010). In contrast, NASH is characterized by severe steatosis, lobular inflammation, and fibrosis of the liver (Powell et al., 1990; Bertot and Adams, 2016). Although the mechanism responsible for the development of NASH remains unclear, NASH is proposed to be caused by a ‘multiple-hit’ process, with hepatic steatosis as the ‘first hit’ and subsequent hits such as inflammation, oxidative stress, and endotoxins (Tilg and Moschen, 2010). NASH is closely related to metabolic syndrome, and several clinical studies have investigated the therapeutic treatment of NASH by focusing on the symptoms of diabetes, hyperlipidemia, and hypertension (Georgescu et al., 2009; Park et al., 2010; Mahady et al., 2011). However, no commonly accepted therapeutic agents have been established.

**FIGURE 1:**
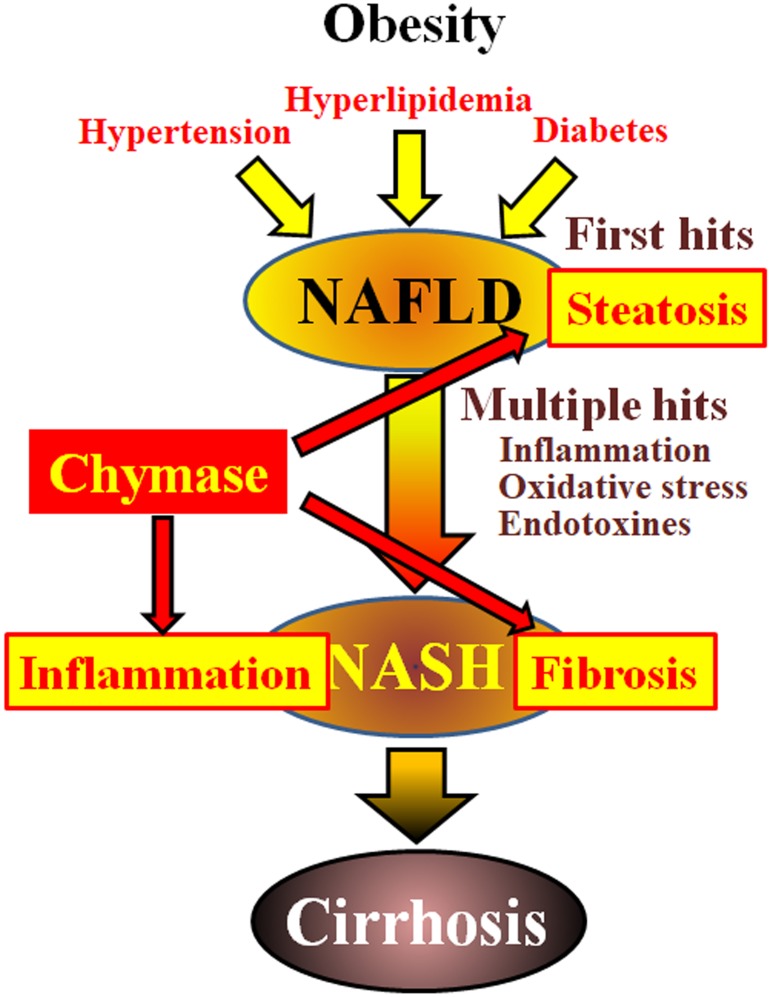
NAFLD and NASH are linked to metabolic syndrome by obesity, insulin resistance, hyperlipidemia, and hypertension. NASH is thought to develop via a ‘multiple-hit’ process, with hepatic steatosis as the “first hit” and subsequent hits such as inflammation, oxidative stress and endotoxins, and is characterized by severe steatosis, inflammation, and fibrosis. Chymase may be involved in the progression of steatosis, inflammation, and fibrosis in liver.

Chymase may be involved in the pathogenesis of hepatic fibrosis. Chymase activity was significantly increased in the livers of patients with fibrosis or cirrhosis and there was a significant correlation between chymase level and degree of fibrosis (Komeda et al., 2008). Although increased chymase activity has not been reported in patients with NASH, it has been observed in animal models of NASH (Tashiro et al., 2010; Masubuchi et al., 2013; Miyaoka et al., 2017). In contrast, the inhibition of chymase using low molecule inhibitors resulted in a significant reduction of inflammation, steatosis, and fibrosis in rat and hamster NASH models (Tashiro et al., 2010; Masubuchi et al., 2013; Miyaoka et al., 2017). These findings indicate that chymase may be involved in inflammation, steatosis, and fibrosis during the development and progression of NASH (**Figure 1**).

## Multipul Functions of Chymase

### Chymase in Mast Cells

**FIGURE 2:**
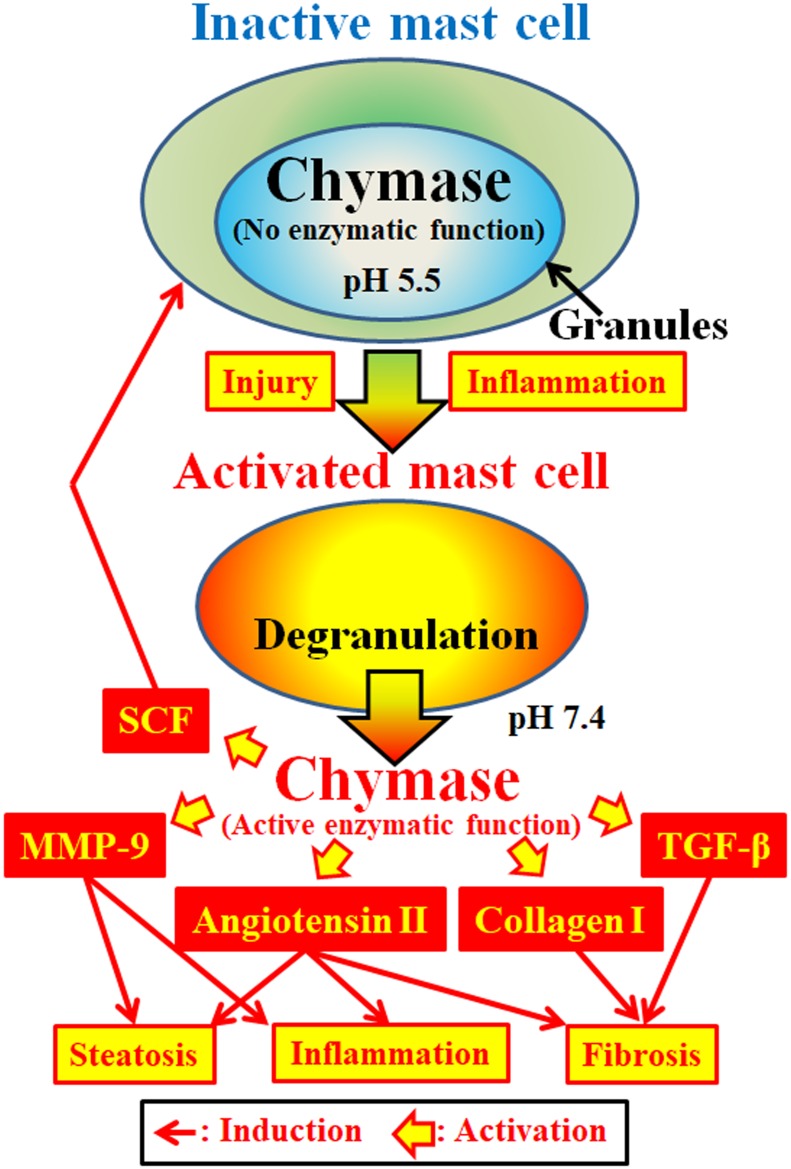
Chymase is stored in the secretory granules of inactive mast cells. The pH within granules is maintained at pH 5.5, a condition in which chymase has no enzymatic activity. Chymase exhibits its enzymatic functions, such as formation of angiotensin II, MMP-9, TGF-β, collagen I and SCF, upon release from mast cell granules, following activation by inflammation and injury.

Chymase (EC 3.4.21.39) is expressed in the secretory granules of mast cells. Chymase is produced as an inactive prochymase within secretory granules, and requires dipeptidyl peptidase I (DPPI) for activation. DPPI is a thiol proteinase and its optimum pH is 6.0. The optimal pH value is consistent with the proposed function of DPPI to activate prochymase, since the pH within secretory granules is regulated at pH 5.5 (De Young et al., 1987) (**Figure 2**). However, chymase has no enzymatic activity within mast cells at this pH, because the optimal pH for chymase is between 7 and 9 (Takai et al., 1996, 1997). Following activation of mast cell granules by stimuli such as inflammation and injury, chymase is released and exhibits enzymatic function at its optimal pH 7.4 (**Figure 2**).

### Multiple Enzymatic Functions of Chymase

Chymase is a serine protease and cleaves the C-terminal side of proteins after aromatic amino acids such as Phe, Tyr, and Trp in general. Chymase can cleave the Phe^8^–His^9^ bond of the non-bioactive peptide angiotensin I and form its bioactive peptide angiotensin II in mammalian tissues including human (Urata et al., 1990; Takai et al., 1996, 1997). In addition, chymase enzymatically cleaves the precursors of matrix metalloproteinase (MMP)-9, transforming growth factor (TGF)-β and collagen I to their active forms (Kofford et al., 1997; Takai et al., 2003; Furubayashi et al., 2008). Furthermore, enzymatic function of chymase can produce stem cell factor (SCF) by enzymatic cleavage of the inactive membrane-bound form of SCF, which induces the formation of mature mast cells from immature mast cells via the stimulation of c-kit receptor (Longley et al., 1997). Thus, chymase has multiple enzymatic functions, including activation of angiotensin II, MMP-9, TGF-β, collagen I, and SCF (**Figure 2**).

### Enzymatic Function of Chymase in NASH

Angiotensin II may promote hepatic steatosis and inflammation by increasing reactive oxygen species (ROS) following stimulation of angiotensin II receptors in animal NASH models (Hirose et al., 2007; Nabeshima et al., 2009). Angiotensin II also induced hepatic fibrosis via induction of α-smooth muscle actin (SMA) in hepatic stellate cells (HSCs) (Yoshiji et al., 2001). MMP-9 has been reported to induce the infiltration of neutrophils and macrophages via degradation of intercellular matrixes such as vitronectin and fibronectin, resulting in augmentation of inflammation (Medina et al., 2006). In NASH patients, a significant increase of MMP-9 gene expression was observed in the liver compared to normal controls (Ljumovic et al., 2004). Hepatic overexpression of TGF-β in transgenic mice produced severe hepatic fibrosis via augmentation of procollagen I gene expression (Casini et al., 1993). Both TGF-β formation and collagen I accumulation are known to induce hepatic fibrosis. Activation of SCF induces increases in mast cell number, and its enzymatic function may result in an increase of chymase activity in fibrotic tissues (Maruichi et al., 2004). These enzymatic functions of chymase may be involved in steatosis, inflammation and fibrosis, all of which are observed in the livers of NASH patients and animal models (**Figure 2**).

### Involvement of Chymase in NASH Animal Models

The methionine- and choline-deficient (MCD) diet has been widely used to induce a typical NASH model. In hamsters fed the MCD diet, significant increases in total bilirubin, triglyceride, and hyaluronic acid were observed in plasma (Tashiro et al., 2010). Moreover, accumulation of inflammatory cells and increases of lipid deposit area and fibrotic area were observed in the liver. In this MCD diet-induced NASH model, hepatic chymase activity and related factors, such as angiotensin II, MMP-9 and collagen I, were significantly increased (Tashiro et al., 2010; Masubuchi et al., 2013). Recently, a new NASH model was developed in which stroke-prone spontaneously hypertensive 5/Dmcr (SHRSP5/Dmcr) rats were fed a high-fat and -cholesterol (HFC) diet (Kitamori et al., 2012). This model showed symptoms of metabolic syndrome thought to clinically resemble those of NASH patients (Kitamori et al., 2012). In the HFC diet-induced NASH model, hypertension and hyperlipidemia were observed, and severe steatosis, fibrosis, and inflammatory cell accumulation were detected in the liver (Miyaoka et al., 2017). Further, a significant augmentation of chymase activity was observed along with MMP-9, TGF-β, and collagen I in the liver (Miyaoka et al., 2017). Thus, there appears to be a close relationship between chymase and NASH pathogenesis in animal models of NASH.

## Effect of Chymase Inhibitor in NASH Animal Models

### Effect of Chymase Inhibitor in NASH Animal Models

A low molecule chymase inhibitor significantly attenuated chymase activity and decreased angiotensin II, MMP-9 and collagen I levels in the liver in an MCD diet-fed NASH hamster model, when administration of the inhibitor was initiated at the same time as the MCD diet (Tashiro et al., 2010; Masubuchi et al., 2013). The chymase inhibitor significantly prevented hepatic steatosis, fibrosis, and inflammatory cell accumulation in this NASH model (Tashiro et al., 2010; Masubuchi et al., 2013). Oxidative stress is thought to play a role in the ‘multiple-hit’ theory of NASH development, and augmentation of the oxidative stress marker malondialdehyde was significantly attenuated in the liver by the chymase inhibitor (Masubuchi et al., 2013). In a hamster MCD diet-induced NASH model, the chymase inhibitor showed an ameliorative effect when administered in established NASH (Masubuchi et al., 2013). The degrees of both steatosis and fibrosis in the liver were reduced compared to before administration of the chymase inhibitor (Masubuchi et al., 2013).

In the liver of a hypertensive rat HFC diet-induced NASH model, a low molecule chymase inhibitor attenuated the levels of chymase as well as MMP-9, TGF-β and collagen I, which are all chymase-associated factors (Miyaoka et al., 2017). The chymase inhibitor significantly attenuated hepatic steatosis and fibrosis, and reduced myeloperoxidase as a marker of inflammation, particularly of neutrophil infiltration (Miyaoka et al., 2017). In this HFC diet-induced model, survival of the placebo-treated group was 0% at 14 weeks following the start of the HFC diet, and resulted from severe liver failure (Miyaoka et al., 2017). However, the chymase inhibitor-treated group, in which the rats were treated with the chymase inhibitor immediately following the start of the HFC diet, showed 100% survival at 14 weeks. Moreover, a 50% survival rate was reported for rats treated with the chymase inhibitor beginning 8 weeks after the start of HFC diet feeding, at which point NASH was established (Miyaoka et al., 2017).

Therefore, chymase inhibitors could be useful agents for the prevention and improvement of NASH in animal models. On the other hand, angiotensin II also indirectly promotes hepatic inflammation, steatosis, and fibrosis via increases of MMP-9 and TGF-β gene expression. Both MMP-9 and TGF-β are closely involved in the pathogenesis of NASH, but these factors are not necessarily induced only by angiotensin II (Takai et al., 2010). Factors other than angiotensin II stimulation contribute to the increases of MMP-9 and TGF-β gene expression (Takai et al., 2010). In such cases, angiotensin II receptor blocker (ARB) is not able to attenuate MMP-9 and TGF-β actions; however, a chymase inhibitor could have attenuating effects via inhibition of MMP-9 and TGF-β activation, indicating a potential treatment course for the prevention of NASH progression.

### Mechanism of Hepatic Inflammation Attenuated by Chymase Inhibitor

Chymase inhibitor was able to reduce inflammation in hamster MCD diet- and rat HFC diet-induced NASH models (Tashiro et al., 2010; Masubuchi et al., 2013; Miyaoka et al., 2017). Chymase inhibitor treatment significantly attenuated chymase activity in the liver as well as reduced angiotensin II and MMP-9 levels (Tashiro et al., 2010; Masubuchi et al., 2013; Miyaoka et al., 2017). In HSC, angiotensin II induces ROS generation such as hydrogen peroxide and superoxide through the activation of nicotinamide adenine dinucleotide phosphate (NADPH) oxidase (De Minicis and Brenner, 2007). Chymase inhibitor resulted in reductions in the gene expression of the NADPH oxidase component Rac-1 and the oxidative stress marker malondialdehyde in addition to a reduction of angiotensin II levels in a hamster MCD-induced NASH model (Masubuchi et al., 2013). Angiotensin II-induced augmentation of ROS promoted MMP-9 gene expression in neutrophils and macrophages (Yaghooti et al., 2011; Kurihara et al., 2012). Therefore, chymase inhibitor directly inhibits the activation of proMMP-9 to MMP-9 and indirectly reduces MMP-9 gene expression via decreased angiotensin II. MMP-9 cleaves extracellular matrix constituents, such as vitronectin and fibronectin, leads to the disintegration of hepatic integrity and induces the infiltration of macrophages and neutrophils (Medina et al., 2006). In a HFC diet-induced NASH model, a significant increase in myeloperoxidase expression in macrophages and neutrophils was observed in the liver, and was reduced by chymase inhibitor (Miyaoka et al., 2017). Therefore, the mechanism of inflammation attenuated by chymase inhibitor may be dependent on the reduction of angiotensin II and MMP-9 levels in the liver.

### Mechanism of Hepatic Steatosis Attenuated by Chymase Inhibitor

Angiotensin II may influence hepatic steatosis via ROS production. In murine HSC, an inhibitor of NADPH oxidase significantly decreased ROS production and an ARB slowed the development of hepatic steatosis via attenuation of ROS production (Hirose et al., 2007; Guimarães et al., 2010). In a MCD diet-induced NASH mouse model, a significant attenuation of steatosis was observed in angiotensin II receptor-deficient mice (Nabeshima et al., 2009). Both *in vivo* and *in vitro* experiments showed that angiotensin II upregulated sterol regulatory element-binding protein (SREBP)-1c and fatty acid synthase (FAS) gene expression, both of which are important factors in the regulation of lipogenesis, following ROS augmentation (Kim et al., 2001; Hongo et al., 2009). In contrast, ARB attenuated hepatic steatosis along with downregulating the gene expression of SREBP-1c and FAS via ROS attenuation in a mouse NASH model (Kato et al., 2012). In a hamster MCD diet-induced NASH model, significant attenuation of SREBP-1c and FAS gene expression was observed following treatment with a low molecule chymase inhibitor (Masubuchi et al., 2013). Therefore, the ameliorative mechanism of hepatic steatosis by chymase inhibitor may be dependent on the reduction of ROS production via reduced angiotensin II generation in the liver.

### Mechanism of Hepatic Fibrosis Attenuated by Chymase Inhibitor

Chymase may be closely associated with the progression of tissue fibrosis, since it contributes to the formation of TGF-β from the non-bioactive precursor TGF-β, and TGF-β is known to strongly induce the growth of fibroblasts (Takai et al., 2003; Oyamada et al., 2011). TGF-β is known to play a central role in the progression of fibrosis in NASH patients via activated HSC (Williams et al., 2000). Inhibition of TGF-β function via gene expression and signaling resulted in improved hepatic fibrosis in experimental models (George et al., 1999; Arias et al., 2003). In a rat HFC diet-induced NASH model, attenuation of chymase activity by chymase inhibitor resulted in reductions in TGF-β level and fibrotic area in the liver (Miyaoka et al., 2017). Thus, the reduction in TGF-β by chymase inhibitor may contribute to the prevention of hepatic fibrosis.

Angiotensin II may also be involved in the induction of hepatic fibrosis. Angiotensin II induces contraction and proliferation of HSC, and also induces the gene expression of TGF-β in fibroblasts *in vitro* (Kagami et al., 1994; Bataller et al., 2000). Both TGF-β levels and the degree of collagen accumulation and fibrotic lesions were observed by bile duct ligation in wild-type mice, however, these were attenuated in angiotensin II receptor-deficient mice (Yang et al., 2005). In a rat NASH model, ARB also attenuated hepatic fibrosis via the reduction of TGF-β gene expression (Hirose et al., 2007). There may also be a relationship between angiotensin II and hepatic fibrosis other than angiotensin II-induced TGF-β gene expression. In patients with chronic hepatitis C, ARB reduced collagen gene expression via Rac-1 gene expression (Colmenero et al., 2009). HSC are recognized as the main producing cells of collagen in the liver, and augmentation in the expression of α-smooth muscle actin (SMA) in HSC strongly induces extracellular matrix deposition, including collagen I (De Minicis and Brenner, 2007). Angiotensin II can induce α-SMA gene expression in rat HSC. In contrast, angiotensin II blockade results in the attenuation of hepatic fibrosis along with reduction of α-SMA (Yoshiji et al., 2001). Although not evaluated in patients with NASH, both chymase and angiotensin II-forming activities were significantly augmented in fibrotic regions of livers from patients with cirrhosis, and significant correlations among chymase, angiotensin II-forming activity and hepatic fibrosis were observed (Komeda et al., 2008). In a hamster tetrachloride-induced hepatic cirrhosis model, significant increases in chymase and angiotensin II-forming activity were observed, which were significantly attenuated along with hepatic cirrhosis following treatment with a low molecule chymase inhibitor (Komeda et al., 2010).

The mast cell stabilizer tranilast could inhibit the activation of mast cells, blocking the release of chymase and thereby preventing the development of hepatic fibrosis in a rat diabetes and HFC diet-induced NASH model (Uno et al., 2008). Chymase promotes the proliferation of mast cells via SCF activation by its enzymatic function (Longley et al., 1997). In NASH animal models, chymase inhibitor reduced the increase in mast cell number in the liver, resulting in reduced chymase activity following direct inhibition by chymase inhibitor and an indirect reduction of chymase expression in mast cells (Masubuchi et al., 2013; Miyaoka et al., 2017).

Therefore, chymase inhibitor may contribute to the prevention of hepatic fibrosis via inhibition of TGF-β activation by chymase inhibition and/or attenuation of TGF-β level via reduction of angiotensin II and mast cell proliferation.

## Conclusion

Metabolic syndrome comprising obesity, insulin resistance, hyperlipidemia, and hypertension is closely related to the development of NASH, and trials of anti-diabetic, anti-hyperlipidemic, and anti-hypertensive agents have been conducted for the treatment of NASH. The concept behind these agents is to attenuate the symptoms of metabolic syndrome (Georgescu et al., 2009; Park et al., 2010; Mahady et al., 2011). Previous reports have demonstrated that chymase inhibitor attenuates inflammation and fibrosis without influencing blood glucose and lipid levels and blood pressure in animal models of diabetes, hyperlipidemia, and hypertension, respectively (Inoue et al., 2009; Takai et al., 2014; Zhang et al., 2016). Therefore, the concept behind chymase inhibition is to attenuate hepatic inflammation and fibrosis of NASH directly. We propose that chymase inhibitor targeting metabolic syndrome is a potentially powerful strategy for the attenuation of NASH progression.

## Author Contributions

ST and DJ: wrote the manuscript. Both authors read and approved the final manuscript.

## Conflict of Interest Statement

The authors declare that the research was conducted in the absence of any commercial or financial relationships that could be construed as a potential conflict of interest.
